# Nonsurgical Endodontic Retreatment of Maxillary Second Molar with Two Palatal Root Canals: A Case Report

**DOI:** 10.5681/joddd.2012.016

**Published:** 2012-06-06

**Authors:** Mahsa Eskandarinezhad, Negin Ghasemi

**Affiliations:** ^1^Assistant Professor, Department of Endodontics, Faculty of Dentistry, Tabriz University of Medical Sciences, Tabriz, Iran; ^2^Post-graduate Student, Department of Endodontics, Faculty of Dentistry, Tabriz University of Medical Sciences, Tabriz, Iran

**Keywords:** Endodontic treatment, human tooth, non-surgical

## Abstract

Successful endodontic treatment requires thorough knowledge regarding each root canal system of any tooth and probability of extra canals should be considered. Second maxillary molar with two palatal root canals is not frequent and its incidence reported in literatures is about 0.4–2%. The present case report describes non-surgical retreatment of maxillary second mo-lar with two palatal root canals. Radiographic interpretation is difficult in this region; so, very careful examination of pulpal space and using supportive devices such as loupe and operating microscope is recommended to discover any unusual anat-omic features like extra canals.

## Introduction

Thorough cleaning, shaping and obturation of entire root canal system are essential tools for successful endodontic treatment. So, a thorough knowledge of root canal morphology and a good anticipation of their possible morphologic variation will help to reduce endodontic failure.^1 - 2^ Unusual root and root canal morphologies associated with molars have been recorded in several studies in the literature.^3 - 5^ Pecora et al evaluated the anatomy of 370 maxillary molars and found that the maxillary first, second, and third molars showed three canals in 75%, 58%, and 68% of the teeth, respectively. Four canals were found in 61.1% of maxillary first molars, in 42% of second molars, and in 32% of third molars. The fourth canal was mainly found in the mesiobuccal root of the teeth.^6^ In the assessment of root canal configuration of maxillary first permanent molars in an Iranian population performed by Shahi et al,^7^ 58.4% of maxillary first molars demonstrated four root canals and in 0.73% of studied first molars there was two palatal root canals. It had been showed that the patient's age was an important predictor of the detection of fewer canals in maxillary molars. This is likely because of the calcification and morphologic changes that occur with age and makes discovery of maximum number of root canals difficult.^8^ This might be one of the reasons of the big discrepancies in the number of detected second, mesiobuccal canals in different studies. Corcoran et al stated that, operator experience has a positive effect on the number of canals located in maxillary molars.^9^ The second maxillary molar usually has one canal in each root, however; it may has two or three mesiobuccal canals, one or two distobuccal canals or two palatal canals. It had been reported that second maxillary molars show two canals in the mesiobuccal roots in up to 58% of the cases.^10^ The frequency of reports on two palatal roots in second maxillary molars is low.^11 - 12^ Slowey first reported maxillary second molars with two palatal roots.^13^ Thews et al^14^ also reported two maxillary second molars with this anatomic variation. Since that time, similar cases have been reported,^14 - 18^ and some attempt has been made to establishing the incidence of double palatal root of maxillary molars. Stone and Stroner examined more than 500 extracted molars and found less than 2% incidence of more than one palatal canal.^19^ Libfeld and Rotstein^20^ reported 0.4% incidence of two palatal canals in an examination of 1000 radiographs and 200 maxillary second molar. Review article by Christie et al^21^ showed that the highest occurrence of two palatal canals in double palatal roots was found in the maxillary second molar. In present case report we reported non-surgical endodontic retreatment of maxillary second molar with two palatal root canals.

## Case report

A 35-years-old male patient without any history of systemic disease was referred to Department of Endodontics at Tabriz University of Medical Sciences, with chief complaint of severe spontaneous pain in the right maxillary second molar. During last six months the endodontic treatment had been performed for this tooth twice, but the patient's symptoms did not relief.

Vitality tests on tooth showed painful response to cold, heat and electrical pulpal test. Tooth response to percussion and palpation was within normal limit. Radiographic evaluation showed pervious obturation of distobuccal and palatal root canals, and the periodontium was normal. Radiographic interpretation was revealed more than one root canal in palatal root (Figure 1a).


Figure 1. (a) Preoperative radiograph of maxillary second molar with two palatal root canals. (b) Radiograph with initial files. (c) Post operative radiograph after treatment.

**a:**
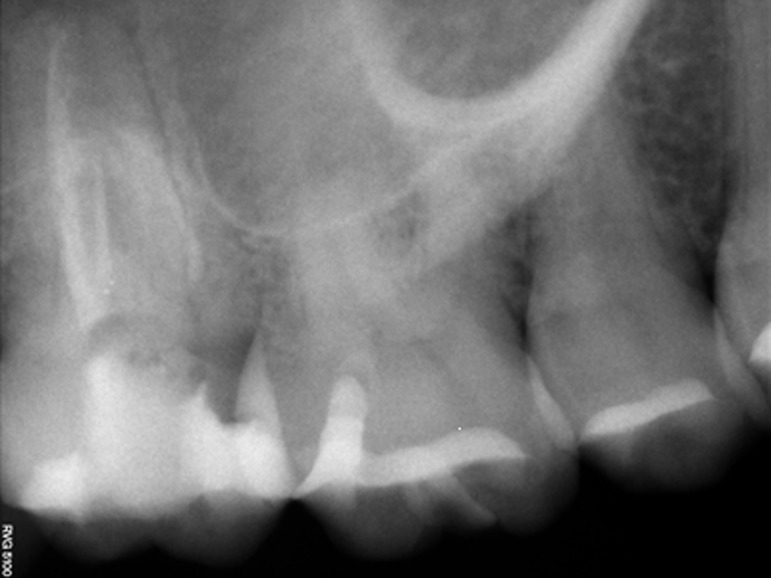

**b:**
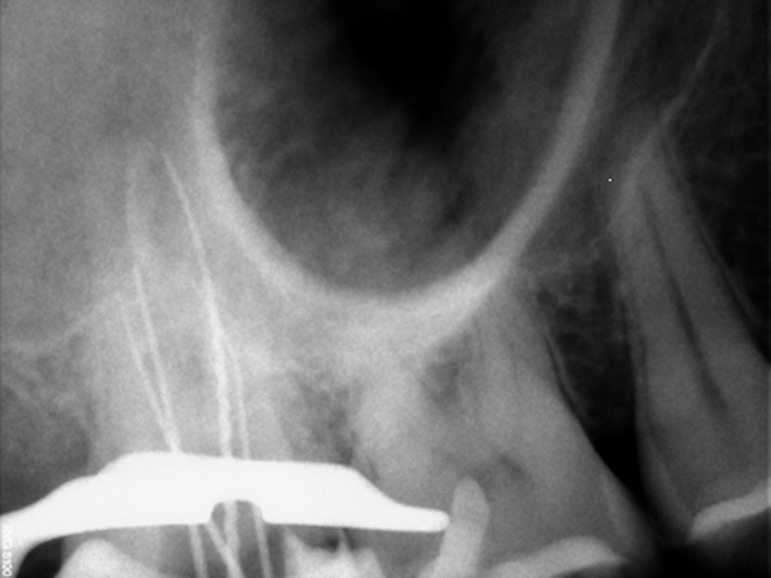

**c:**
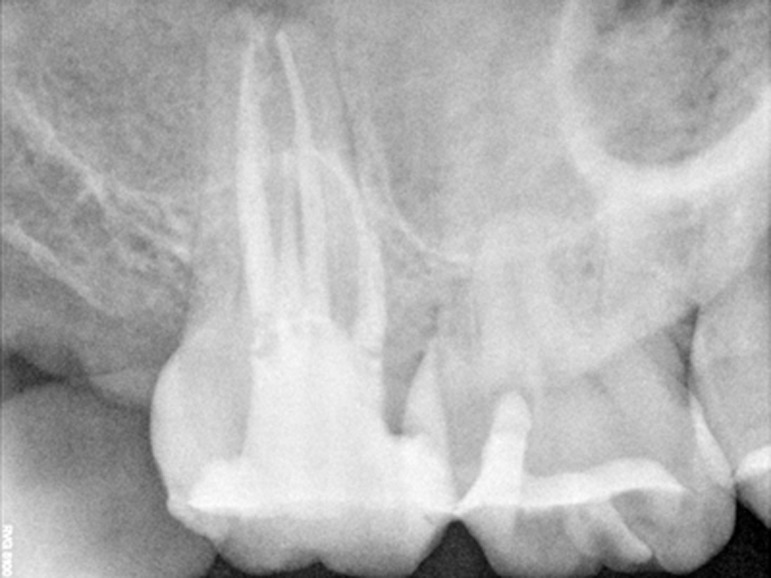

Non-surgical endodontic retreatment was planned for #2 tooth. The patient was anesthetized with 2% lidocaine and 1:80,000 epinephrine. After isolation with rubber dam, access to the pulp chamber was made. Four canal orifices were found and confirmed via visualization under operating microscope (OPMI pico Dental Microscope, Zeiss, Oberkochen, Germany). Gutta-percha was removed using rotary files. Working length was established with the use of an apex locator (Root ZX, J. Mortina Inc, USA) and confirmed by a radiograph (Figure 1b). The canals were cleaned and shaped with hand k-files (Maillefer Dentisply, Baillaigues, Switzerland) and RaCe NiTi rotary files in crown down manner up to final canal size #0.06/30 in palatal canals and #0.06/25 in buccal canals. The canals were irrigated with 2.5% sodium hypochlorite during instrumentation and 17% EDTA at the end of instrumentation. After final rinse with normal saline, canals were dried and obturated with Gutta-percha and AH-26 sealer (Dentsply, De Trey, Konstanz, Germany) using the lateral compaction method (Figure 1c). Then, the patient was referred to restorative department.

## Discussion

Knowledge of pulp anatomy is essential for success of endodontic treatment, and lack of such knowledge may lead to treatment failure.^22^

The occurrence of second maxillary molars with two palatal roots or two palatal root canals is not frequent and its incidence reported in literatures is about 0.4-2%.^15 , 21^ It is interesting that the majority of molars with two palatal canals are second maxillary molars rather than first ones.^21^

Since the preparation of radiographic images of second molars with high quality is more difficult, one should be aware that the anomaly occurs more frequently in this region.^21^ Careful evaluation of X-rays is required to detect morphological variation of maxillary second molar. An unusually massive coronal morphology should also attract the attention of the dentist during the clinical examination.^15^ Using magnifier loup, fiber optic illumination for observation of anatomical land marks in the pulp chamber, sodium hypochlorite bubbling in the extra canals and dyes may be helpful in locating additional canals.^23^ Furthermore, cone beam computed tomography was described as a valuable method for initial identification of the internal or external morphology.^24- 25^ Evaluation of CBCT images always resulted in a greater number of root canal systems than the previously mentioned techniques.^26 - 27^ In our case the presence the second palatal canal was obvious, but for better visualization we used operating microscope.

Christie et al have proposed classification system for four-rooted maxillary molars.^21^ Type I maxillary molars have two widely divergent palatal roots that are often long. The buccal roots of these teeth are often 'cow-horn' shaped and less divergent. Four separate root apices are seen in the radiograph. A type II maxillary molar has four separated roots also but the roots are often shorter, run parallel, have buccal and lingual root morphology and have blunt root apices. A radiograph with buccolingual superimposition may make this type appear as having only a mesial and distal root. A type III maxillary molar is also constricted in root morphology with the mesiobuccal, mesiopalatal and distopalatal canal encaged in a web of root dentin. Type II and III are difficult to identification by radiography alone. The case presented in this report was type III according to this classification.

Teeth with two palatal roots often seem to have wider mesiodistal dimension over the palatal cusp, so the access should be wider in lingual than usual and the access outline take on a square shape than triangular.^16^ Periodontal and root probing placed in these cases before the ruuber dam, will often help in determining the morphology of root trunk.^15^ The prognosis of treatment these teeth should be similar to any molar endodontic prognosis.^21^

## Conclusion

Anatomic variations can occur in many teeth, and palatal root of maxillary second molar is not exception; so, the clinician should not focus only on the variations of mesiobuccal root in the maxillary molars and careful radiographic evaluation and clinical examination of pulp chamber with magnification should be considered.
